# Relationship between IL-8 Circulating Levels and TLR2 Hepatic Expression in Women with Morbid Obesity and Nonalcoholic Steatohepatitis

**DOI:** 10.3390/ijms21114189

**Published:** 2020-06-11

**Authors:** Teresa Auguet, Laia Bertran, Jessica Binetti, Carmen Aguilar, Salomé Martínez, Fàtima Sabench, Jesús Miguel Lopez-Dupla, José Antonio Porras, David Riesco, Daniel Del Castillo, Cristóbal Richart

**Affiliations:** 1 Grup de Recerca GEMMAIR (AGAUR)-Medicina Aplicada (URV), Departament de Medicina i Cirurgia, Institut d’Investigació Sanitària Pere Virgili (IISPV), Universitat Rovira i Virgili (URV), 43007 Tarragona, Spain; laia.bertran@urv.cat (L.B.); jessica.binetti@gmail.com (J.B.); caguilar.hj23.ics@gencat.cat (C.A.); jmlopezdupla.hj23.ics@gencat.cat (J.M.L.-D.); aporras.hj23.ics@gencat.cat (J.A.P.); cristobalmanuel.richart@urv.cat (C.R.); 2 Hospital Universitari de Tarragona Joan XXIII, Servei Medicina Interna, 43007 Tarragona, Spain; driesco.hj23.ics@gencat.cat; 3 Hospital Universitari de Tarragona Joan XXIII, Servei Anatomia Patològica, 43007 Tarragona, Spain; mgonzalez.hj23.ics@gencat.cat; 4 Hospital Universitari Sant Joan de Reus, Servei de Cirurgia, Departament de Medicina i Cirurgia, Institut d’Investigació Sanitària Pere Virgili (IISPV), Universitat Rovira i Virgili (URV), 43204 Reus, Spain; fatima.sabench@urv.cat (F.S.); danieldel.castillo@urv.cat (D.D.C.)

**Keywords:** cytokines, toll-like receptors, morbid obesity, nonalcoholic fatty liver disease, nonalcoholic steatohepatitis

## Abstract

The progression of nonalcoholic fatty liver disease (NAFLD) to nonalcoholic steatohepatitis (NASH) is linked to systemic inflammation. Currently, two of the aspects that need further investigation are diagnosis and treatment of NASH. In this sense, the aim of this study was to assess the relationship between circulating levels of cytokines, hepatic expression of toll-like receptors (TLRs), and degrees of NAFLD, and to investigate whether these levels could serve as noninvasive biomarkers of NASH. The present study assessed plasma levels of cytokines in 29 normal-weight women and 82 women with morbid obesity (MO) (subclassified: normal liver (*n* = 29), simple steatosis (*n* = 32), and NASH (*n* = 21)). We used enzyme-linked immunosorbent assays (ELISAs) to quantify cytokine and TLR4 levels and RTqPCR to assess TLRs hepatic expression. IL-1β, IL-8, IL-10, TNF-α, tPAI-1, and MCP-1 levels were increased, and adiponectin levels were decreased in women with MO. IL-8 was significantly higher in MO with NASH than in NL. To sum up, high levels of IL-8 were associated with the diagnosis of NASH in a cohort of women with morbid obesity. Moreover, a positive correlation between TLR2 hepatic expression and IL-8 circulating levels was found.

## 1. Introduction

Nonalcoholic fatty liver disease (NAFLD) comprises a wide spectrum of liver cell injuries that range from simple steatosis (SS) to nonalcoholic steatohepatitis (NASH) [1,2]. NASH is the severe form of NAFLD and, if left untreated, can evolve into end-stage liver diseases such as cirrhosis and hepatocellular carcinoma [3]. The risk of liver-related mortality increases exponentially with an advance of the fibrosis stage [4]. NAFLD is a major public health concern because of its increased prevalence worldwide, affecting 6–45% of the general population; however, up to 90% of patients with morbid obesity (MO) develop NAFLD [5]. Obesity and NAFLD share common pathogenic factors, such as insulin resistance [6,7], and obesity per se is considered an independent risk factor for NAFLD [8].

**Figure 1:**
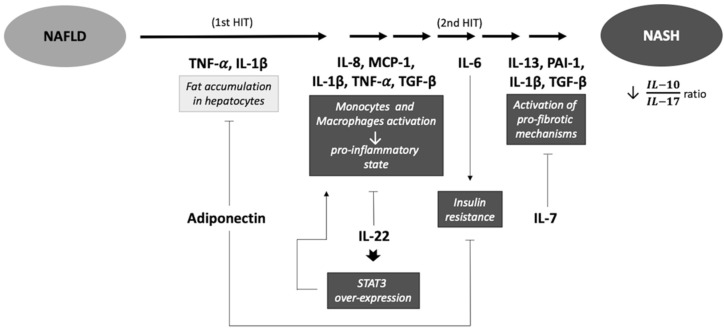
Pro- and anti-inflammatory cytokines involved in the physiopathology of NAFLD progression. IL-1β promotes fat accumulation in hepatocytes and then, it induces liver inflammation and stimulates liver fibrosis in a late stage of the disease [21,31]; TGF-β performs the same function [29]. TNF-α mediates the early stage of NAFLD by fat accumulation in hepatocytes and also facilitates disease progression to a more advanced stage. IL-8 and MCP-1 activate monocytes and macrophages and attract polymorphonuclear leukocytes to the site of inflammation inducing a proinflammatory microenvironment [22,32]. IL-6 induces insulin resistance in hepatocytes [23]. IL-13 appears to activate distinct profibrotic mechanisms during the progression of NAFLD [24]. PAI-1 is a primary regulator of the fibrinolytic system in NASH [25]. A more accentuated proinflammatory stage in NASH patients is indicated by a decrease of the IL-10/IL-17 ratio [26]. IL-7 is known to suppress expression of several molecules known to promote fibrosis and stimulates the expression of factors that protect against it [27]. Adiponectin regulates hepatic insulin resistance and is related to hepatic fat content [22,33]. IL-22 has a dual role in hepatic inflammation; this cytokine has a protective effect against liver damage inducing hepatocyte repair, but it also has a proinflammatory and tumorigenic role in the liver because of the overexpression of STAT-3 induced by a successive induction of IL-22 [28,34]. NAFLD, nonalcoholic fatty liver disease; NASH, nonalcoholic steatohepatitis; IL-1β, interleukin-1β; TNF-α, tumor necrosis factor-α; IL-8, interleukin-8; MCP-1, monocyte chemoattractant protein-1; IL-6, interleukin-6; IL-13, interleukin-13; PAI-1, plasminogen activator inhibitor-1; IL-10, interleukin; IL-17, interleukin-17; IL-7, interleukin-7; IL-22, interleukin-22; TGF-β, transforming growth factor beta; STAT-3, signal transducer and activation of transcription-3.

Adipocyte hypertrophy causes a deregulation of the expression of adipokines and alterations of immune system function. The result is an obesity-associated low-grade systemic inflammation that is characterized by the recruitment of macrophages and increased levels of proinflammatory cytokines and chemokines, as well as the activation of several kinases and transcription factors that regulate inflammation and insulin sensitivity in adipocytes and hepatocytes [9,10]. The mechanism of NAFLD progression is usually explained with the classic “multiple hit” theory of NAFLD pathogenesis, which states that lipid accumulation initiates hepatic steatosis [11,12,13] and subsequently triggers multiple insults (adipokines secretion, inflammation, lipotoxicity, deregulation in the glucose and lipids metabolism, and so forth), finally inducing NASH and cirrhosis. The progression to NASH is linked to systemic inflammation, and it is associated with other pathological processes, such as innate immunity alterations, endoplasmic reticulum stress, mitochondrial dysfunction, toll-like receptors (TLRs) signaling, and intestinal dysbiosis [11,14,15,16,17,18]. Adipocytokines are mediators in many of these processes, and adipocytokine deregulation leads to an activation of the immune system, a shift to the proinflammatory M1-phenotype of macrophages, and activation of hepatic stellate cells [17,19]. These changes contribute to a vicious cycle that perpetuates the inflammatory response and hepatocyte damage [20]. Previous clinical studies have reported that several cytokines are involved in the physiopathology of NAFLD progression. On the one hand, circulating levels of some proinflammatory cytokines seem to be directly related to NAFLD progression, such as interleukin (IL)-1β, tumor necrosis factor (TNF)-α, IL-8, monocyte chemoattractant protein (MCP)-1, IL-6, IL-13, plasminogen activator inhibitor (PAI)-1, and IL-17 [21,22,23,24,25,26]. On the other hand, circulating anti-inflammatory cytokines (IL-7, IL-10, and adiponectin) are diminished in NASH [22,26,27]. Moreover, IL-22 has a dual role in NAFLD progression because it can act as an anti-inflammatory or a proinflammatory cytokine [28]. Studies have addressed the role of transforming growth factor beta (TGF-β) in inflammation and fibrosis, where in the liver TGF-β is a major modulator of disease progression [29]. The extensive inflammation associated with cytokine/chemokine release ultimately leads to the accumulation of extracellular matrix, the development of fibrosis, and further deterioration of liver function [30] (Figure 1).

Lipotoxicity and oxidative stress in hepatocytes produced by lipid accumulation in NAFLD induce a massive release of proinflammatory cytokines that promotes the progression to NASH [35,36,37]. However, this fact was only demonstrated by limited human experimental studies, and except for the NASH Clinical Research Network Study [25], most of them were published as small clinical data and results cannot be extrapolated to a larger cohort of study [22,38,39,40].

Proinflammatory cytokines might be increased in NAFLD in relation to the activation of TLRs in the liver. TLRs are a family of pattern-recognition receptors that play a key role in the activation of the innate immune system by recognizing pathogen-associated molecular patterns (PAMPs). There is an increased intestinal permeability associated with the progression of NASH: proinflammatory low-grade chronic state related to NASH promotes tight junction damage. This increased permeability of the intestinal barrier triggers a massive translocation of bacterial components, such as LPS or bacterial DNA, among others. When these PAMPS reach the liver through the portal vein circulation, they activate the TLRs and induce IL production in Kupffer cells. This ultimately triggers the progression of NASH [41]. Several studies in animal models have demonstrated that TLR2, 4, and 9 play a fundamental role in the development of NASH. The lack of these receptors in diet-induced NASH mice has been shown to lead to a lower immune response that slows down the progression of NASH than in wild-type mice models [42,43,44]. Probably, TLRs and cytokines act together in NASH pathogenesis, through intestinal dysbiosis [45].

Moreover, the research activity in therapies for NAFLD is currently very intense because, although there are available drugs for other indications with beneficial effects for these patients, there is no approved pharmacological treatment for NASH. In this sense, it has been demonstrated that blocking several cytokines with monoclonal antibodies significantly attenuates liver fibrosis and inflammation and may have a potential therapeutic effect in patients with NASH and/or liver fibrosis. Specifically, CCL24, a chemokine that regulates inflammation and fibrosis, was found to be significantly expressed in patients with NASH, in whom it regulates profibrotic processes in the liver. Also, the blockade of CCL24 using a monoclonal antibody robustly attenuated liver fibrosis and inflammation in animal models, thus suggesting a potential therapeutic role for an anti-CCL24 agent [46].

Presently, liver biopsy is still required to identify patients with NASH and early fibrosis. However, its invasive nature, high cost, and inter- and intraobserver variability make it less suitable for diagnosis and disease monitoring in clinical practice and unsuitable for screening at a population level [47,48,49]. Moreover, fibrosis panels, such as FIB4, that cannot distinguish between fatty liver and steatohepatitis, should not be used to diagnose NASH [50].Therefore, it is essential to search for noninvasive soluble biomarkers with robust analytical and clinical validation for diagnosing NASH and fibrosis severity.

Although the implication of inflammatory cytokines in NAFLD and NASH is well documented, the cytokine role in the progression of the disease, possible cytokine therapeutic targets, or cytokine efficacy in the diagnosis and follow-up of NASH are not well established. Therefore, in the present project, we had the following objectives: first, we sought to study circulating levels of cytokines and soluble TLR4 in a cohort of biopsy-proven NAFLD women with morbid obesity (MO) in relation to the hepatic expression of TLRs (TLR2, TLR4, and TLR9). Next, we sought to assess whether the circulating levels of cytokines are associated with the severity of the disease and can be used as noninvasive biomarkers of NASH.

## 2. Results

### 2.1. Baseline Characteristics of Subjects

The clinical characteristics and biochemical measurements of the population studied are shown in Table 1. We classified the patients into two groups according to their body mass index (BMI): normal-weight (NW) women (BMI < 25 kg/m^2^; *n* = 29) and women with MO (BMI > 40 kg/m^2^; *n* = 82). Biochemical analyses indicated that women with MO had significantly higher levels of fasting glucose (*p* < 0.001), insulin (*p* < 0.001), glycosylated hemoglobin (HbA1c) (*p* = 0.032), homeostatic model assessment method insulin resistance (HOMA)2-IR (*p* < 0.001), and triglycerides (TGL) (*p* < 0.001) than NW women. High-density lipoprotein cholesterol (HDL-C) (*p* < 0.001) was significantly lower in patients with MO than NW women. Levels of aspartate aminotransferase (AST) (*p* = 0.008), alanine aminotransferase (ALT) (*p* < 0.001), gamma-glutamyl transferase (GGT) (*p* < 0.001), and alkaline phosphatase (ALP) (*p* < 0.001) were higher in the group with MO.

We also classified the cohort of patients with MO according to liver pathology into normal liver (NL, *n* = 29), simple steatosis (SS, *n* = 32) and nonalcoholic steatohepatitis (NASH, *n* = 21). Fasting glucose (*p* < 0.001) and ALP (*p* = 0.006) activity were significantly higher in SS patients than in the NL. ALP (*p* = 0.008) activity was significantly higher in NASH patients than in the SS group.

### 2.2. Circulating Levels of Adipocytokines in the Population Studied

We determined the plasma levels of IL-1β, IL-6, IL-7, IL-8, IL-22, IL-13, IL-10, IL-17, TNF-α, tPAI-1, MCP-1, and adiponectin in NW women and women with MO. In five cases, the determination of adiponectin had been considered laboratory measurement errors. These determinations were eliminated because there was not enough sample available to repeat them. Circulating IL-1β, IL-8, IL-10, TNF-α, tPAI-1, and MCP-1 levels were increased in women with MO, and circulating adiponectin levels were decreased in this group (Table 2). However, our results indicated no significant differences between the plasma levels of IL-6, IL-7, IL-22, or IL-17 between NW women and patients with MO.

**Figure 2:**
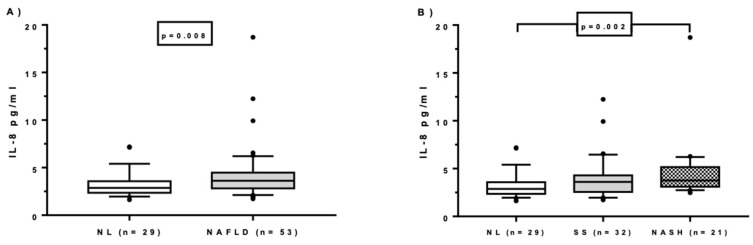
Circulating levels of IL-8 in women with morbid obesity with normal liver and with nonalcoholic fatty liver disease (**A**), and women with morbid obesity according to liver damage (**B**). NAFLD, women with morbid obesity (MO) with nonalcoholic fatty liver disease; NL, women with MO with normal liver; SS, women with MO with simple steatosis; NASH, women with MO with steatohepatitis. *p* < 0.05 is considered statistically significant.

Analyses of the circulating levels of adipocytokines in MO patients revealed that IL-8 and adiponectin were differentially expressed in plasma samples. Specifically, IL-8 levels were significantly higher in women with MO with NAFLD than without NAFLD (Figure 2A).

To examine the possible role of cytokines in NAFLD, we further divided NAFLD patients into SS and NASH. We observed that significant differences in IL-8 levels were found between the NL and NASH groups (Figure 2B).

There were no differences in the circulating levels of the other cytokines or TLR4 between groups (Table 3).

### 2.3. Correlations between Circulating Cytokine Levels, TLR4 Levels, and TLRs Hepatic Expression

A positive correlation between IL-8 and other cytokines was found in the entire cohort: IL-1β (rho = 0.416, *p* < 0.001), IL-6 (rho = 0.436, *p* < 0.001), and TNF-α (rho = 0.511, *p* < 0.001). In the obese cohort, a positive correlation between IL-8 and other cytokines was also found with IL-1β (rho = 0.249, *p* = 0.03), IL-6 (rho = 0.335, *p* = 0.003), and TNF-α (rho = 0.394, *p* < 0.01).

Given the relationship of TLRs and cytokines in the pathogenesis of NASH, we also explored the association between circulating levels of cytokines, TLR4 levels, and the hepatic expression of TLR2, TLR4, TLR9 in the liver. We only found a positive correlation between TLR2 hepatic expression and IL-8 circulating levels (rho = 0.257, *p* = 0.046) in the obese cohort.

### 2.4. Circulating Levels of Cytokines and Histopathological Features

**Figure 3:**
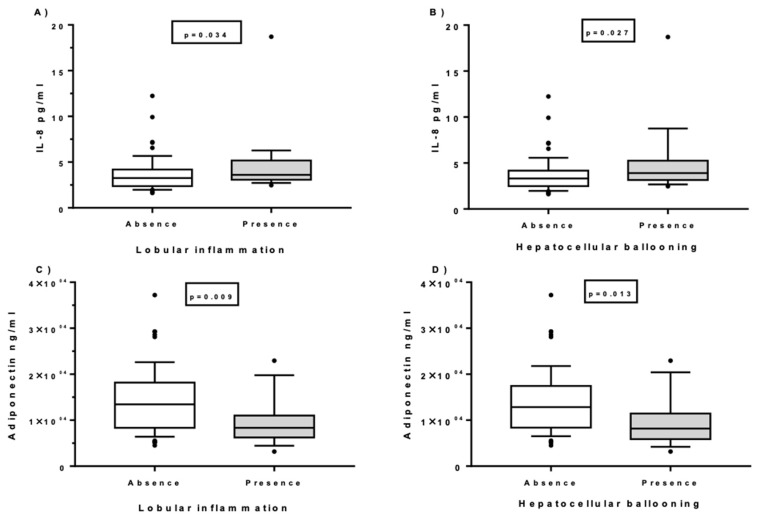
Circulating levels of IL-8 and adiponectin in women with MO according to the presence or absence of lobular inflammation (**A**,**C**) and hepatocellular ballooning (**B**,**D**). Data are expressed as medians (10–90th). *p* < 0.05 is considered statistically significant.

Circulating levels of IL-8 were positively associated with the presence of lobular inflammation and hepatocellular ballooning whereas adiponectin levels were negatively associated with lobular inflammation and hepatocellular ballooning (Figure 3). No other associations were found between the levels of IL-1β, IL-6, IL-7, IL-22, IL-10, IL-13, IL-17, TNF-α, tPAI-1, or MCP-1 with histopathological parameters.

### 2.5. Evaluation of Circulating Cytokine Levels as Biomarkers of Nonalcoholic Steatohepatitis

**Figure 4:**
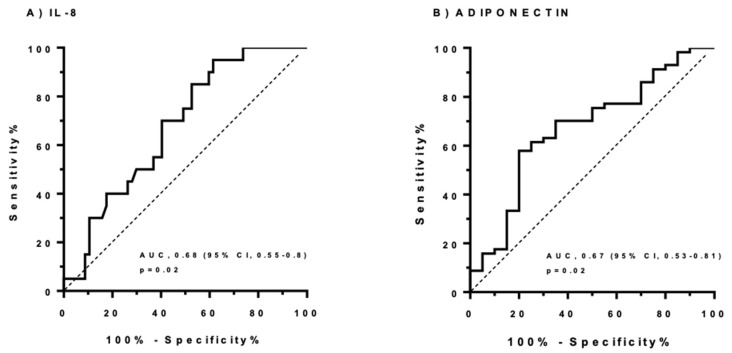
Receiver operating characteristic (ROC) curves for NASH diagnosis. (**A**) ROC curve for the association between plasma IL-8 levels (pg/mL) and NASH diagnosis in MO patients. (**B**) ROC curve for the association between plasma adiponectin levels (ng/mL) and no NASH diagnosis in MO patients. The dashed line is a reference line indicating chance prediction. AUC denotes area under the curve.

As a final step, we evaluated the diagnostic efficacy of circulating IL-8 and adiponectin levels as markers of NASH in a group of patients with liver histology indicative of NASH. A cut-off point and area under the curve were determined so that NASH could be diagnosed. To evaluate the extent to which these cytokines can predict histological features, a receiver operating characteristic (ROC) curve was obtained. The accuracy with which this panel discriminates NASH subjects from non-NASH subjects in the case of IL-8 showed an area under the ROC curve (AUROC) of approximately 0.68 (Figure 4A). On the contrary, plasma adiponectin levels were predictors of no NASH, with an AUROC of 0.67 (Figure 4B).

## 3. Discussion

In the present study, we examined the status of circulating cytokines in women with MO and their possible association with the diagnosis and progression of NAFLD in relation with TLR hepatic expression. We found that most of the analyzed cytokines (IL-1β, IL-8, IL-10, TNF-α, tPAI-1, MCP-1, and adiponectin) were associated with obesity. However, only IL-8 was correlated to the presence of NAFLD in women with MO. Finally, a positive correlation between TLR2 hepatic expression and IL-8 circulating levels was found.

First, we analyzed the relationship of cytokine levels to obesity. We found increased levels of plasma IL-1β, IL-8, IL-10, TNF-α, tPAI-1, and MCP-1 in women with MO compared to NW subjects. These results are similar to reports published previously, which found that increased levels of proinflammatory cytokines were related to obesity [40,51,52,53,54,55]. We also found decreased levels of adiponectin in women with morbid obesity, which is consistent with the literature [39]. This result is explained by the obesity-related low-grade systemic inflammation, in which adipose tissue dysfunction, hyperplasia, and hypertrophy of adipocytes causes a hypersecretion of cytokines and chemokines, which are linked to stimulation of the immune system, activation of intracellular proinflammatory cascades, and diverse transcription factors and immune cell recruitment, which perpetuate the proinflammatory circulating profile in subjects with obesity [52,54,55,56].

Second, in an attempt to assess whether a proinflammatory cytokine profile was related to the diagnosis and progression of NAFLD, we analyzed whether plasma adipocytokine levels differed between NAFLD and NL in the cohort of women with MO. It is known that inflammation is a crucial event in the occurrence of NAFLD and the progression from SS to NASH. In fact, inflammatory cytokines, such as TNF-α, IL-1β, IL-6, and IL-8, have been studied in patients with NAFLD [22,25,47,57,58]. In our study, we found that IL-8 was the only cytokine with higher levels in NAFLD compared to NL. Previous studies obtained similar results in patients with NAFLD compared to control subjects [40,59,60] and patients with NASH compared to SS patients [25,61]. Lipid accumulation in hepatocytes induces production of IL-8. This interleukin is increased in obese individuals with IR and was previously associated with steatosis degree and lobular inflammation [22]. Serum IL-8 in Hispanic pediatric patients with obesity correlated with the hepatic fat fraction [62].

Not only cytokines, but a variety of other markers have been evaluated in liver diseases including liver enzymes, matrix components, or caspase-cleaved K18 fragments (M30) [29,56]. In NAFLD, an extensive body of literature has also looked at cytokines [18,25,31]. In our study, several cytokines did not show significant differences between NAFLD and NL patients. Probably, the inflammatory process underlying obesity could mask the results of circulating cytokine levels in these two morbidly obese groups, independently of NAFLD presence.

Although the role of cytokines in NASH remains controversial in humans, the evidence in animal models is clearer. Some studies have shown that TNF-α knock-out mice [63] or IL-1-deficient mice [64] with diet-induced NASH trigger a milder and slower NASH progression than wild-type mice models. Moreover, the use of monoclonal antibodies inhibiting chemokines such as CCL24 reduced NASH progression [46]. Given the importance of cytokines in NASH progression, designing novel therapeutic approaches by their modulation has even been recently considered [65].

Finally, we explored the surrogate association between cytokine plasma levels and the histopathological features of NASH in the cohort of women with MO. IL-8 and adiponectin were associated with the presence of lobular inflammation and hepatocellular ballooning in our cohort of patients. Previous published studies demonstrated that IL-8 was a significant discriminator of NASH severity because of its associations with advance of steatosis degree [57], with steatosis degree and lobular inflammation [14,66], fibrosis [39], hepatocyte ballooning and significant fibrosis [25], and cirrhosis [67]. In the present study, adiponectin levels were negatively correlated with lobular inflammation and hepatocellular ballooning. There are reports of adiponectin being a good predictor of the necro-inflammatory grade and fibrosis in NAFLD via mechanisms that were clarified in vitro [68]. In our work, we could not study the relationship with fibrosis because all of our patients with NASH were evaluated histologically for the presence of fibrosis and none of them presented this histological feature. This fact could be explained in part because our cohort was made up of middle-aged women with no other cause of liver disease than obesity and insulin resistance. However, other possible anti-inflammatory adipocytokines, such as IL-10, IL-13, and IL-22, did not correlate with histopathological liver features in our study.

Based on our results, we included circulating IL-8 and adiponectin levels as markers of NASH in a group of patients with liver histology indicative of NASH. In regard to IL-8, the AUROC obtained was 0.68. Meanwhile, plasma adiponectin levels were predictors of no NASH, with an AUROC of 0.67. Unfortunately, the results obtained showed that they are not good biomarkers on their own.

To sum up, certain cytokines, such as IL-8 or adiponectin, may determine the severity of NAFLD histology [22].

Of particular interest among our findings is the relationship between IL-8 circulating levels and TLR2 hepatic expression. Currently, little data exist regarding TLR2 and NAFLD. However, there is evidence that TLR2-mediated pathways crucially contribute to the progression of NAFLD/NASH [69]. Moreover, Miura et al. showed that TLR2 knock-out mice models have a slower NASH progression than wild-type mice models [44]. Taken together, our results may suggest a possible role of TLR2 in the pathogenesis of NASH, in relation with IL-8.

Some limitations should be considered. First, the cross-sectional methodology of the study did not offer information about the causal link between these two adipocytokines and liver damage. Second, we investigated a cohort of women with MO with different degrees of liver histology. Because of these limitations, the data cannot be extrapolated to other obesity or overweight groups or men. Also, our research has no cohort validation, so these results must be validated in large, population-based studies. In this sense, further studies are needed to progress in clarifying NAFLD pathogenesis, identifying therapeutic targets, and advancing in drug development. Also, the validation of predictive noninvasive biomarkers of disease risk and response to treatment is necessary [70].

## 4. Materials and Methods

### 4.1. Study Subjects

The institutional review board “Comitè d’Ètica d’Investigació Clínica, Hospital Universitari Joan XXIII de Tarragona” approved the study (23c/2015). All participants gave written informed consent for their participation in medical research. We included 111 Caucasian women: 82 women with morbid obesity (MO) (BMI > 40 kg/m^2^) and 29 normal-weight (NW) control women (BMI < 25 kg/m^2^). Liver biopsies were only obtained from women with morbid obesity during planned bariatric surgery and were always performed for diagnostic indications. The diagnosis of NAFLD was made based on the following criteria: (1) liver pathology, (2) an intake of less than 10 g of ethanol/day, and (3) the exclusion of other liver diseases. The following exclusion criteria were used: (1) subjects who had an alcohol consumption higher than 10 g/day or other toxins; (2) patients who had an acute or chronic hepatic or inflammatory disease, infectious disease, or neoplastic disease; (3) menopausal women or women using contraceptives; (4) diabetic women receiving pioglitazone, GLP-1 receptor agonists, DPP-4 inhibitors, or insulin; and (5) patients under steatotic drug treatment.

### 4.2. Sample Size

Accepting an α risk of 0.05 and a β risk of less than 0.2 in a bilateral contrast, 24 subjects per group are needed to detect a difference ≥0.2 units. It is assumed that the common standard deviation is 0.3.

### 4.3. Liver Pathology

Experienced hepatopathologists scored liver samples using methods described elsewhere [71,72]. Simple steatosis (SS) was graded as follows: Grade 1 or mild SS: greater than 5% and less than 33% of hepatocytes affected; Grade 2 or moderate SS: 33% to 66% of hepatocytes affected; or Grade 3 or severe SS: more than 66% of hepatocytes affected. The minimum criteria for the steatohepatitis diagnosis included the presence of either ballooning cells and lobular inflammation or perisinusoidal/pericellular fibrosis in zone 3 of the hepatic acinus. None of the patients with NASH in our cohort presented fibrosis. Histological parameters in NAFLD patients, such as lobular inflammation and hepatocellular ballooning, were dichotomized into absent or present for further comparisons.

Women with morbid obesity were subclassified into three groups based on liver pathology: normal liver (NL) histology (*n* = 29); simple steatosis (SS) (micro/macrovesicular steatosis without inflammation or fibrosis, *n* = 32); and nonalcoholic steatohepatitis (NASH) (Brunt Grades 1–3, *n* = 21).

### 4.4. Biochemical Analyses

All subjects, the women with MO and control group, underwent physical, anthropometrical, and biochemical assessments. Blood samples were obtained from women with MO and control subjects. Biochemical parameters were measured after overnight fasting. Insulin resistance (IR) was estimated using the homeostasis model assessment of IR (HOMA2-IR).

Plasma samples were stored at −80 °C. Cytokines, such as IL-1β, IL-6, IL-7, IL-8, TNF-α, adiponectin, tPAI-1, and MCP-1, were determined using multiplex sandwich immunoassays and the MILLIPLEX MAP Human Adipokine Magnetic Bead Panel 1 (HADK1MAG-61K, Millipore, Billerica, MA, USA) and MILLIPLEX MAP Human High-Sensitivity T Cell Panel (HSTCMAG28SK, Millipore, Billerica, MA, USA), and the Bio-Plex 200 instrument at the Center for Omic Sciences (Universitat Rovira i Virgili), according to the manufacturer’s instructions. Circulating levels of IL-13, IL-17, IL-10, and IL-22 (Quantikine, R & D Systems, Minneapolis, MN, USA) were measured in duplicate using enzyme-linked immunosorbent assays (ELISAs) following the manufacturer’s instructions. TLR4 levels were analyzed by enzyme-linked immunosorbent assay (ELISA) according to the manufacturer’s instructions (Ref. SEA753Hu; USCN).

### 4.5. Gene Expression in the Liver

Liver samples were collected during bariatric surgery and were conserved in RNAlater (Qiagen, Hilden, Germany) at 4 °C and then processed and stored at −80 °C. Total RNA was extracted from both tissues by using the RNeasy mini kit (Qiagen, Barcelona, Spain). Reverse transcription to cDNA was performed with the High-Capacity RNA-to-cDNA Kit (Applied Biosystems, Madrid, Spain). Real-time quantitative PCR (RTqPCR) was performed with the TaqMan Assay predesigned by Applied Biosystems for the detection of TLR2, TLR4, TLR9 in the liver. The expression of each gene was calculated relative to the expression of 18S RNA. All reactions were carried out in triplicate in 96-well plates using the 7900HT Fast Real-Time PCR system (Applied Biosystems, Madrid, Spain).

### 4.6. Statistical Analysis

The data were analyzed using SPSS/PC+ for Windows statistical package (version 23.0; SPSS, Chicago, IL, USA). The Kolmogorov–Smirnov test was used to assess the distribution of variables. Continuous demographic, clinical, and laboratory measures are reported as means ± SD. Plasma cytokines are reported as medians and 25–75th percentiles, and categorical variables are shown as counts (percent). Different comparison analyses were performed using Student’s test and one-way ANOVA with Bonferroni post hoc test for parametric variables and Mann–Whitney’s U test or Kruskal–Wallis test for nonparametric variables, according to the presence of two or more groups. The strength of the association between variables was calculated using Spearman’s rho correlation test (nonparametric variables). *p*-values < 0.05 were statistically significant. The area under the receiver operating characteristic curve (AUROC) was used as an accuracy index for evaluating the diagnostic performance of the selected variables.

## 5. Conclusions

Increased levels of IL-8 were associated with the diagnosis of NASH in a cohort of women with morbid obesity. Moreover, a positive correlation between TLR2 hepatic expression and IL-8 circulating levels was found.

## Figures and Tables

**Table 1:** Anthropometric and metabolic variables of study cohort classified according to BMI and histopathological characteristics.

Variables	NW (*n* = 29)	MO (*n* = 82)	NL (*n* = 29)	SS (*n* = 32)	NASH (*n* = 21)
Age (years)	41.99 ± 9.20	46.31 ± 10.78	43.05 ± 10.35	47.49 ± 11.54	48.99 ± 9.45
Weight (kg)	57.01 ± 6.26 *	118.19 ± 16.10	119.10 ± 19.88	119.81 ± 13.94	114.45 ± 13.23
BMI (kg/m^2^)	21.56 ± 2.17 *	44.92 ± 5.03	44.38 ± 5.34	45.63 ± 5.42	44.57 ± 3.93
GLUC (mg/dL)	81.03 ± 6.79 *	109.57 ± 60.97	91.86 ± 42.51 **^§^**	135.15 ± 82.11	95.04 ± 18.79
INS (mUI/L)	6.15 ± 1.83 *	15.93 ± 14.30	11.98 ± 8.68	19.36 ± 18.03	16.61 ± 14.23
HOMA2-IR	0.78 ± 0.23 *	2.08 ± 1.87	1.54 ± 1.10	2.61 ± 2.42	2.10 ± 1.76
HbA1c (%)	5.34 ± 0.37 *	6.00 ± 1.17	5.63 ± 0.72	6.42 ± 1.50	5.92 ± 1.00
COL (mg/dl)	180.88 ± 33.74	175.39 ± 36.65	172.60 ± 35.49	173.55 ± 35.54	181.11 ± 40.64
HDL-C (mg/dL)	71.30 ± 13.47 *	41.85 ± 11.45	41.89 ± 10.84	43.96 ± 13.62	38.55 ± 7.84
LDL-C (mg/dL)	96.15 ± 28.20	103.79 ± 28.64	107.74 ± 27.33	100.90 ± 29.24	103.06 ± 30.59
TGL (mg/dL)	64.88 ± 27.92 *	139.92 ± 70.17	114.36 ± 31.56	141.23 ± 59.13	167.73 ± 102.07
AST (U/L)	18.80 ± 5.15 *	28.50 ± 17.37	26.22 ± 14.72	26.73 ± 15.72	33.95 ± 21.88
ALT (U/L)	17.50 ± 7.45 *	30.81 ± 17.79	27.71 ± 15.34	32.19 ± 16.97	32.90 ± 21.89
GGT (U/L)	15.56 ± 8.12 *	29.18 ± 28.71	27.32 ± 30.85	31.74 ± 32.09	27.74 ± 19.08
ALP (U/L)	54.15 ± 13.24 *	67.45 ± 15.53	62.15 ± 14.90 **^§^**	75.00 ± 15.35 **^#^**	62.59 ± 12.36

NW, normal-weight; MO, morbid obesity; NL, normal liver; SS, simple steatosis; NASH, nonalcoholic steatohepatitis; BMI, body mass index; GLUC, glucose; INS, insulin; HOMA2-IR, homeostatic model assessment method insulin resistance; HbA1c, glycosylated hemoglobin; COL, cholesterol; HDL-C, high-density lipoprotein cholesterol; LDL-C, low-density lipoprotein cholesterol; TGL; triglycerides; AST, aspartate aminotransferase; ALT, alanine aminotransferase; GGT, gamma-glutamyl transferase; ALP, alkaline phosphatase. Insulin resistance was estimated using homeostasis model assessment of IR (HOMA2-IR). Data are expressed as the means ± SD. * Significant differences between NW controls and women with MO (*p* < 0.05). ^§^ Significant differences between NL and SS (*p* < 0.05). ^#^ Significant differences between SS and NASH (*p* < 0.05).

**Table 2:** Circulating levels of cytokines and TLR4 in women with morbid obesity and normal-weight subjects.

Variables	NW (*n* = 29)	MO (*n* = 82)	*p*-Value
IL-1β (pg/mL)	2.85 (2.36–4.03)	4.12 (2.93–5.93)	0.004
IL-6 (pg/mL)	3.90 (2.57–6.09)	3.63 (2.42–5.73)	0.439
IL-7 (pg/mL)	6.53 (4.64–9.02)	6.60 (4.45–8.95)	0.788
IL-8 (pg/mL)	2.92 (1.81–3.46)	3.44 (2.67–4.29)	0.010
IL-22 (pg/mL)	2.69 (0.35–10.27)	3.81 (0.46–9.11)	0.669
IL-13 (pg/mL)	7.11 (1.31–10.78)	5.15 (2.00–22.64)	0.390
IL-10 (pg/mL)	1.33 (1.01–2.97)	3.28 (1.51–6.49)	<0.001
IL-17 (pg/mL)	0.19 (0.04–0.37)	0.23 (0.02–0.28)	0.158
TNF-α (pg/mL)	6.59 (4.77–7.65)	10.25 (7.68–12.22)	<0.001
tPAI-1(ng/mL)	13.02 (7.97–18.62)	62.96 (44.09–101.15)	<0.001
MCP-1 (pg/mL)	47.59 (38.70–57.28)	85.95 (66.49–110.49)	<0.001
Adiponectin (ng/mL)	20,146.39 (9800.70–24,305.90)	11,774.80 (7504–17,049.80)	0.007
TLR4 (ng/mL)	2.80 (1.88–4.23)	2.63 (1.61–3.26)	0.152

NW, normal-weight; MO, morbid obesity; IL-1β, interleukin 1b; IL-6, interleukin 6; IL-7, interleukin 7; IL-8, interleukin 8; IL-22, interleukin 22; IL-13 interleukin 13; IL-10, interleukin 10; IL-17, interleukin 17; TNF-α, tumor necrosis factor alpha; tPAI-1, total plasminogen activator inhibitor 1; MCP-1, monocyte chemoattractant protein-1; Adiponectin; TLR4, toll-like receptor 4. Data are expressed as medians (25–75th). *p* < 0.05 is considered statistically significant.

**Table 3:** Circulating levels of cytokines and TLR4 in the group with morbid obesity according to liver histology.

Variables	NL (*n* = 29)	SS (*n* = 32)	NASH (*n* = 21)	*p*-Value
IL-1β (pg/mL)	3.59 (2.94–5.48)	4.38 (2.53–6.33)	4.47 (3.35–7.21)	0.538
IL-6 (pg/mL)	3.99 (2.62–5.78)	3.11 (2.33–4.41)	4.16 (2.34–6.33)	0.255
IL-7 (pg/mL)	6.46 (3.76–9.17)	6.15 (4.52–8.25)	7.39 (4.97–12.03)	0.633
IL-8 (pg/mL)	2.87 (2.35–3.58) ^¤^	3.61 (2.56–4.29)	3.75 (3.11–5.16)	0.013
IL-22 (pg/mL)	2.34 (0.35–7.73)	3.85 (1.40–11.13)	3.84 (0.35–8.15)	0.326
IL-13 (pg/mL)	5.15 (1.35–20.37)	5.06 (2.01–21.19)	6.682 (2.41–28.37)	0.641
IL-10 (pg/mL)	3.72 (1.40–6.92)	2.65 (1.78–4.59)	3.36 (1.44–8.34)	0.960
IL-17 (pg/mL)	0.11 (0.02–0.28)	0.25 (0.02–0.33)	0.25 (0.02–0.28)	0.890
TNF-α (pg/mL)	10.15 (8.01–11.75)	9.94 (7.22–11.44)	11.11 (8.71–12.57)	0.510
tPAI-1 (ng/mL)	59.26 (40.11–84.80)	68.99 (47.19–117.70)	66.86 (37.77–101.41)	0.227
MCP-1 (ng/mL)	81.99 (54.57–97.84)	99.48 (76.31–115.62)	74.08 (61.24–103.72)	0.130
Adiponectin (ng/mL)	13,807.08 (8189.6–19,471.4) ^¤^	12,698.42 (8114.50–16,986.45)	8496.70 (6431.6–11,659)	0.060
TLR4 (ng/mL)	2.62 (1.88–3.06)	1.99(0.84–2.97)	2.72(1.70–5.26)	0.670

IL-1β, interleukin 1b; IL-6, interleukin 6; IL-7, interleukin 7; IL-8, interleukin 8; IL-22, interleukin 22; IL-13 interleukin 13; IL-10, interleukin 10; IL-17, interleukin 17; TNF-α, tumor necrosis factor alpha; tPAI-1, total plasminogen activator inhibitor 1; MCP-1, monocyte chemoattractant protein-1; Adiponectin; TLR4, toll-like receptor 4. Data are expressed as medians (25–75th). ^¤^ Significant differences between NL and NASH (*p* < 0.05).

## Abbreviations

NAFLD	Nonalcoholic fatty liver disease
SS	Simple steatosis
NASH	Nonalcoholic steatohepatitis
NW	Normal-weight
MO	Morbid obesity
NL	Normal liver
TLR2	Toll-like receptor 2
TLR4	Toll-like receptor 4
TLR9	Toll-like receptor 9
BMI	Body mass index
HOMA2-IR	Homeostatic model assessment method-insulin resistance
HbA1c	Glycosylated hemoglobin
HDL-C	High-density lipoprotein cholesterol
LDL-C	Low-density lipoprotein cholesterol
AST	Aspartate aminotransferase
ALT	Alanine aminotransferase
GGT	Gamma-glutamyl transferase
ALP	Alkaline phosphatase
IL-1β	Interleukin 1beta
IL-6	Interleukin 6
IL-7	Interleukin 7
IL-8	Interleukin 8
IL-10	Interleukin 10
IL-13	Interleukin 13
IL-17	Interleukin 17
IL-22	Interleukin 22
TNF-α	Tumor necrosis factor alpha
tPAI-1	Total plasminogen activator inhibitor 1
MCP-1	Monocyte chemo attractant protein 1
TGF-β	Transforming growth factor beta
